# The feasibility and safety of combining atrial septal defect/patent foramen ovale and left atrial appendage closure: A systematic review and meta-analysis

**DOI:** 10.3389/fcvm.2022.1080257

**Published:** 2023-01-06

**Authors:** Yi Song, Hang Xing, Peter David Koch, Xiaofei Li, Yan Zhang

**Affiliations:** ^1^Department of Ultrasound, The First Affiliated Hospital of Zhengzhou University, Zhengzhou, Henan, China; ^2^Department of Surgery, Rhode Island Hospital, Warren Alpert Medical School of Brown University, Providence, RI, United States; ^3^Center for Systems Biology, Massachusetts General Hospital, Boston, MA, United States; ^4^Division of Cardiology, Department of Medicine, Cardiovascular Research Center, Rhode Island Hospital, Warren Alpert Medical School of Brown University, Providence, RI, United States

**Keywords:** atrial appendage closure/occlusion, atrial septal closure/occlusion, patent foramen ovale closure/occlusion, atrial fibrillation, staged

## Abstract

**Introduction:**

Atrial Septal Defect/Patent Foramen Ovale (ASD/PFO) occlusion is performed to prevent paradoxical embolism and reduce the risk of recurrent ischemic stroke. Left atrial appendage (LAA) closure is used as an alternative to medical therapy of non-valvular atrial fibrillation for prevention of stroke. Multiple studies have examined performing LAA and ASD/PFO occlusion. However, the feasibility and safety of combined occlusion of the left atrial appendage and ASD/PFO are not clear, furthermore, these studies are limited by their small sample sizes and retrospective analysis. In this study, we aimed to systematically review and meta-analyze the feasibility and safety of combining left atrial appendage and ASD/PFO closure.

**Methods:**

PubMed, Web of Science, CNKI, Cochrane Library, Embase, and WanFang database were searched up to April 2022 to identify peer-reviewed human studies on assessing the feasibility, safety, and efficacy of combining left atrial appendage and ASD/PFO closure. The primary outcome was calculated: procedural feasibility outcome and procedural safety outcome.

**Results:**

A total of 10 articles, including 340 patients from multiple countries, were included in the analysis. The principal findings of our study are: compared with single LAA closure, (i) combining PFO/ASD occlusion and LAA closure had similar procedural success proportion (98.43%, 95% CI: 96.67–100.00%), (ii) similar safety event incidences developed (1.67%, 95% CI: 0.24–3.92%), subgroup analyzed safety event incidences in death was 0.00 (95% CI: 0.00–0.33%), cardiac tamponade was 0.87% (95% CI: 0.00–2.77%), device embolization was 0.00 (95% CI: 0.00–0.60%), major bleeding was 0.00 (95% CI: 0.00–0.33%), stroke was 0.00 (95% CI: 0.00–0.02%).

**Conclusion:**

Although this systematic review and meta-analysis demonstrate the technical feasibility and safety of combining closure of PFO/ASD and LAA, further studies of sufficient sample size, long-term follow-up, and rigor endpoint criteria are yet needed to fully evaluate this combination procedure for its role in clinical outcomes.

## 1. Introduction

Patent foramen ovale (PFO) and Atrial septal defect (ASD) are among the most common congenital heart malformations, they are the deficiencies in the septum separating the two atrial chambers. PFO is found in almost a quarter of the general population, which is the consequence of failed closure of the foramen ovale after birth (1). PFO has been considered as an important factor in cryptogenic stroke; the responsible mechanism has been attributed to paradoxical embolism that shunts through the PFO to the cerebral circulation. ASD shows an incidence of about 0.1% in livebirths and 30% in adults suffering from congenital heart defects. ASD allows shunting between the systemic and the pulmonary circulations, leading to right atrial pressure elevation and right atrial enlargement, thus developing the common complications of ASD such as heart failure, atrial fibrillation (AF). In AF, the blood pools and becomes sluggish, which can result in the formation of blood clots and increase the risk of stroke.

Medical interventions for the complications (e.g., stroke and AF) of PFO/ASD, such as antiplatelet and anticoagulant therapies, have limitations and side-effects. Cardiac implant closure is an efficient alternative to the patients who cannot tolerate the medications. However, patients without AF still have a higher risk for developing AF after ASD closure than that of the general population (2), and the risk increases after PFO closure in the presence of enlarged atria (3). Left atrial appendage (LAA) closure is used as an alternative to medical therapy of non-valvular AF for prevention of stroke. The rationale behind its use is that 90% of the thrombi originate from left atrial appendage (4). LAA closure shows significantly improved survival in randomized comparisons (5), the occurrence of iatrogenic atrial septal defects is common after LAA closure, but the iatrogenic atrial septal defects are not associated with an increased rate of stroke/systemic embolization during long-term follow-up.

Based on the current expert consensus, combining PFO/ASD and LAA closure should be limited to rather specific cases. LAA closure is currently recommended in patients with non-valvular AF who are not candidates for anticoagulation and at high thrombo-embolic risk (CHA2DS2- VASc ≥ 2) (class IIb, level of evidence B) (6, 7), however, the most widely recognized indication for PFO closure is embolic stroke of unknown source or in patients with large shunts due to PFO, which can only be diagnosed by ruling out other sources of stroke such as AF; carotid dissection; intracerebral pathology; and atherosclerotic disease (8, 9). Thus, from the current clinical guidelines, patients with AF who are not candidates for anticoagulation and also have a PFO with prior stroke may undergo LAA occlusion but they should not have their PFO closed. ASD closure is indicated in the presence of a significant left-to-right shunt, defined by a significant right heart enlargement due to volume overload, regardless of symptoms (10–12), therefore patients with secundum ASD and AF who are not candidates for anticoagulation may have closure of the LAA and ASD.

Despite these situations are rare, multiple studies involved performing combining LAA and PFO/ASD closure. However, the feasibility and safety of combining left atrial appendage and PFO/ASD closure are not clear due to the limited number of patients. In this study, we pooled available evidence and data of closing both the LAA and PFO/ASD in a systematic review and meta-analysis to assess the feasibility and safety of this combining procedure.

## 2. Methods

### 2.1. Eligibility criteria

Peer-reviewed studies were included, provided they were in accordance with the following criteria: (1) the procedural success, safety and efficacy outcome of combining percutaneous left atrial appendage and PFO/ASD closure and (2) sufficient published data for calculating relative proportion risk. When the articles were associated with the same population, the most appropriate article that followed the above criteria was selected. The exclusion criteria were review articles or single case reports.

### 2.2. Literature search

The literature search with no language restriction was completed by using PubMed, Web of Science, CNKI, Cochrane Library, Embase, WanFang database (published before April 2022). Search terms were used as following: “atrial appendage closure”, “atrial septal closure,” “patent foramen ovale closure.” Animal studies were excluded. Two reviewers independently evaluated all titles and abstracts. Inconsistent assessment between reviewers was resolved by discussion. If the uncertainty still exists after discussion, a third reviewer evaluated the article to resolve the discrepancy. After excluding articles based on the title and abstracts, the remaining research articles were studied.

### 2.3. Data extraction

Data extraction using a standardized form was done by two reviewers to collect relevant data from all included studies systematically. The obtained data included the mean age of patients, the number of included patients, sex distribution, history of disease, follow-up duration, post-procedure antithrombotic regimen, LAA device and PFO/ASD device brand. Methodologic quality was assessed by two reviewers using the modified Newcastle-Ottawa scale (NOS) for each candidate study. Disagreements between reviewers were resolved by discussion.

### 2.4. Outcome measures

The primary outcome of this meta-analysis was procedural feasibility and safety. Procedural feasibility was the proportion of patients who met the procedural success criteria of the study defined or the proportion of procedural success patients mentioned in the study. Procedural safety was the proportion of patients that experienced periprocedural complications (death, pericardial effusion, device embolization, major bleeding, major access vessel complication, and stroke) and other relevant complications leading to prolonged hospital stay.

The secondary outcome of this meta-analysis was procedural efficacy, which was the proportion of patients that experienced all-cause stroke, cardiovascular and unexplained deaths, and systemic embolism.

### 2.5. Reference standard

We searched a meta-analysis of LAA closure or PFO/ASD closure on Pubmed as a reference standard to compare with the outcome of our study. However, we failed to select a meta-analysis of PFO/ASD closure with enough data to analyze. Finally, we selected Yerasi et al. as reference standard of procedural feasibility and safety outcome (13), Yerasi’s meta-analyses focused on single LAA closure.

### 2.6. Statistical analysis

The R (version 4.0.5) software package was performed for Meta-analysis. The *I*^2^ statistic was used to assess inconsistency in results across studies. The *I*^2^ statistic quantifies the amount of variability across studies, which is due to the true differences in design, tests, patients, and outcomes rather than chance. The values 75, 50, and 25% indicate high, moderate, and low statistical heterogeneity, respectively. To evaluate the source of heterogeneity, subgroup analyses were performed. Egger’s test was applied to evaluate publication bias. The decision flow chart for the selection of the statistical model in this meta-analysis was previously described (14). The flow chart has some criteria, including the goal of statistical inference, the number of studies in the meta-analysis, and statistical heterogeneity.

## 3. Results

### 3.1. Study selection

**FIGURE 1:**
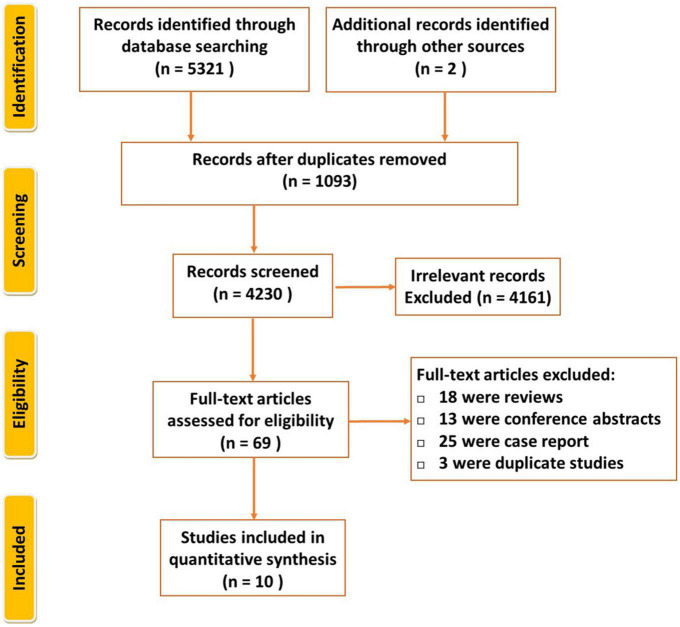
Flowchart for selection of the included studies.

PRISMA flow diagram is shown in Figure 1. By the initial literature search and additional records identified through other sources, 5,323 relevant articles were identified, then 4,230 records were composed after duplicates removed. We read all titles and abstracts and selected 69 articles for reviewing in detail. Fifty-nine studies were excluded because they were either abstracts, review articles, or case reports, duplicate. Finally, ten articles fulfilled the eligibility criteria (3, 15–23).

### 3.2. Study characteristics

The selected studies encompassed 340 patients with a mean age of 66.6 years old. The sample size of the included studies ranged from 6 to 133 participants. The sample included patients who originated from multiple countries including Switzerland, Germany, China, and Malaysia. Seven of them were single-center studies (15, 17–19, 21–23), and three were multiple-center studies (3, 16, 20). The watchman LAA device was used in three studies, and the Amplatzer LAA device was used in four studies (15, 16, 18, 20). PLAATO and LACBES LAA devices were used in Gafoor et al. and Wang et al. (15, 19), respectively. Amplatzer PFO/ASD devices were used in four studies (3, 15, 17, 21), SHSMA PFO/ASD devices were used in Wang et al. (19), while five studies did not mention which brand of PFO/ASD occluder used (16, 18, 20, 22, 23). The width of LAA was evaluated by angiography and transesophageal echocardiography; the width of the ASD/PFO was evaluated by balloon sizing, transesophageal echocardiography, or both. Table 1 and Supplementary Table 1 details information about the patient recruitment, post procedure antithrombotic regimen, follow-up duration, and other clinical characteristics. The patient selection criteria was shown in Supplementary Table 2.

**TABLE 1:** Clinical characteristics.

References	Number of patients	Age at first intervention (mean ± SD)	Gender (m/f)	CHA_2_DS_2_-VASc (mean ± SD)	HAS-BLED score (mean ± SD)	NOS score
Koermendy et al. (17)	51	71 ± 12	35/16	2.5 ± 1.4	2.4 ± 1.3	7
Gafoor et al. (15)	17	63.5 ± 9.8	**–**	3.9 ± 1.1	–	5
Wang et al. (19)	18	56.3 ± 6.9	8/10	2.4 ± 0.8	3.1 ± 0.5	7
Kuwata et al. (3)	13	58.2 ± 9.1	6/7	1.92 ± 1.7	0.9 ± 1.1	7
Yu et al. (21)	30	75.4 ± 7.3	26/4	3.8 ± 1.6	3.7 ± 1.1	7
Kleinecke et al. (20)	133	73.3 ± 10.8	**–**	4.2 ± 1.7	2.7 ± 1.1	7
Leong et al. (18)	6	68.6 ± 0.8	2/4	3.8 ± 0.8	1.8 ± 0.8	7
Jiang et al. (16)	13	64.8 ± 3.9	5/8	3.2 ± 0.3	2.2 ± 0.3	7
Zhang et al. (22)	49	65.6 ± 9.6	22/27	3.5 ± 0.8	2.6 ± 0.6	7
Jiang et al. (23)	10	69.4 ± 9.2	7/3	3.8 ± 1.4	2.6 ± 0.7	7

### 3.3. Synthesis of results

**FIGURE 2:**
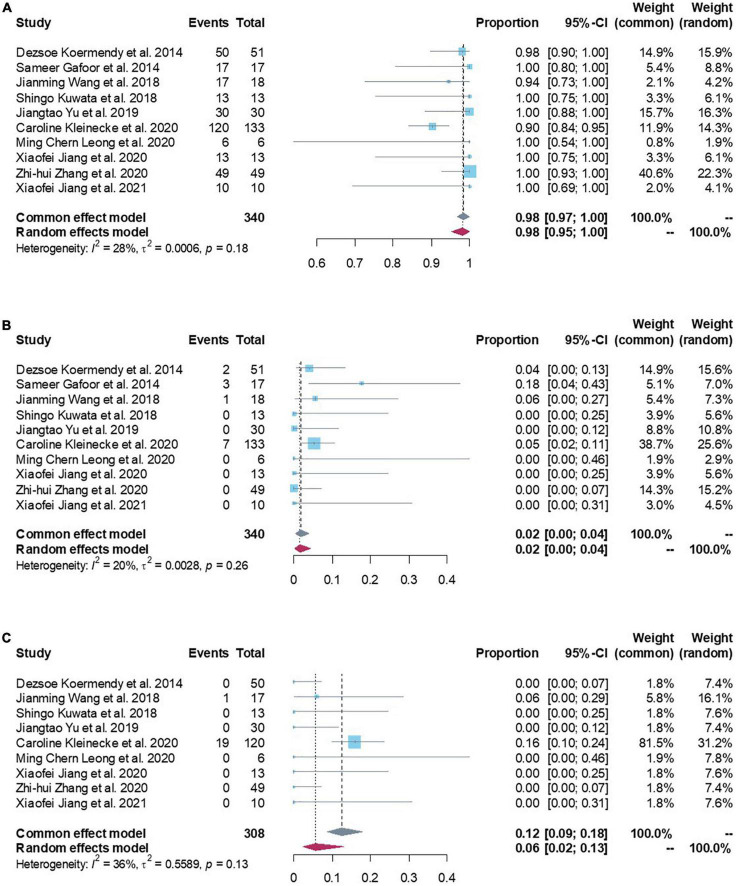
**(A)** Forest plots of procedural feasibility outcome (procedural success proportion). **(B)** Forest plots of procedural safety outcome (death/device embolization/cardiac tamponade/major bleeding/stroke/major access vessel complication/other). **(C)** Forest plots of procedural efficacy outcome (all-cause stroke/systemic embolism/cardiovascular and unexplained deaths).

The successful combining LAA and PFO/ASD closure from all studies contained enough data to perform a proportion meta-analysis. Low heterogeneity between the studies was found with an inconsistency (*I*^2^) of 28% (*p* = 0.18). Consequently, a fixed model was finally chosen. Results from the meta-analysis showed overall successful proportion of 98.43% (95% CI: 96.67–100.00%) from a total sample of 340 patients, which was not significantly different with the reference standard (Figure 2A and Table 2). Egger’s test indicated no publication bias in this meta-analysis.

**TABLE 2:** Subgroup analyses of procedural feasibility and safety outcome.

		The presence study		The reference study		*p*-value
		Mean (%)	95% confidence intervals (%)	Mean (%)	95% confidence intervals (%)	
Procedural success		98.43	96.67–100.00	96.20	95.90–96.50	0.6742
Procedural safety outcome	Death	0.00	0.00–0.33	0.20	0.10–0.30	0.517
	Cardiac tamponade	0.87	0.00–2.77	1.20	1.00–1.40	0.6324
	Device embolisation	0.00	0.00–0.60	0.10	0.30–0.50	0.747
	Major bleeding	0.00	0.00–0.33	1.20	0.90–1.40	0.1209
	Stroke	0.00	0.00–0.02	0.30	0.20–0.40	0.3304

Procedural safety events were reported in all studies with 340 patients. Low heterogeneity was found between the studies (*I*^2^ of 20%; *p* = 0.26). Meta-analysis results showed a procedural safety events proportion of 1.67% (95% CI: 0.24–3.92%), suggesting that patients had a lower safety events rate in peri-procedure of combined LAA and PFO/ASD closure (Figure 2B). Egger’s test indicated no publication bias in this meta-analysis. Subgroup analyzed all safety events by fixed effect model, the results showed pooled death was 0.00 (95% CI: 0.00–0.33%), cardiac tamponade was 0.87% (95% CI: 0.00–2.77%), device embolization was 0.00 (95% CI: 0.00–0.60%), major bleeding was 0.00 (95% CI: 0.00–0.33%), stroke was 0.00 (95% CI: 0.00–0.02%; Table 2), which were not significantly different with the reference standard.

Procedural efficacy events were reported in nine studies with 308 patients (3, 16–23). Moderate heterogeneity was found between the studies (*I*^2^ of 36%; *p* = 0.13). Meta-analysis results showed a procedural efficacy events proportion of 12.40% (95% CI: 8.59–17.90%, Figure 2C). Egger’s test indicated no publication bias in this meta-analysis. Subgroup analyzed all efficacy events by fixed effect model, the results showed pooled all-cause stroke was 2.19% (95% CI: 0.88–5.44%), systemic embolism was 2.18% (95% CI: 0.96–4.97%), cardiovascular and unexplained deaths was 8.50% (95% CI: 5.51–13.10%; Table 3).

**TABLE 3:** Subgroup analyses of procedural efficacy outcome.

		Mean (%)	95% confidence intervals (%)
Procedural efficacy outcome	All-cause stroke	2.19	0.88–5.44
	Systemic embolism	2.18	0.96–4.97
	Cardiovascular and unexplained deaths	8.50	5.51–13.10

## 4. Discussion

Current guidelines recommend percutaneous PFO/ASD closure for prevention of stroke or congenital heart disease. LAA closure is used to prevent stroke in patients with AF. Multiple randomized controlled trials show the procedure of PFO/ASD closure or LAA closure were commonly performed and relatively safe. However, the occlusion of the left atrial appendage and PFO/ASD combined is not clear. Meanwhile, the feasibility, safety and efficacy of performing PFO/ASD closure and LAA closure was not evaluated in larger scale study. We included appropriate relevant studies to conduct this systematic review and meta-analysis to assess the combining of PFO/ASD occlusion and LAA closure. The principal finding of this study was that: compared with single LAA closure, combining PFO/ASD occlusion and LAA closure had similar procedural success proportion, and similar procedural safety event incidence during the peri-procedural period.

**FIGURE 3:**
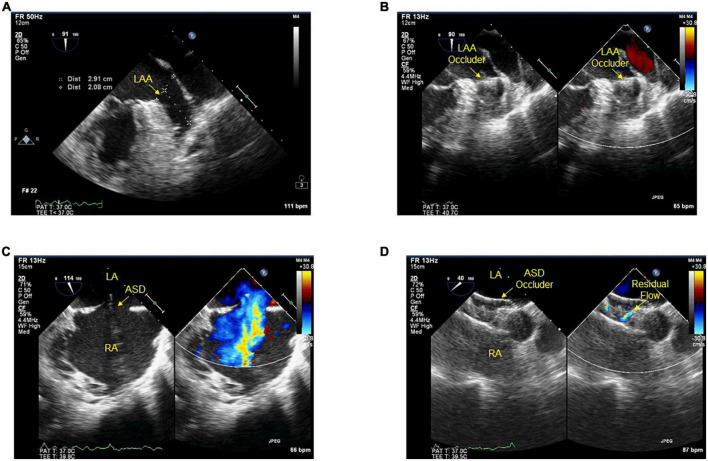
**(A–D)** Transesophageal echocardiography during procedures. Before **(A)** and after **(B)** the device implanted into the ostium of LAA. Before **(C)** and after **(D)** ASD closed with the device, residual flow ≤2 mm around the device after implantation. LAA, left atrial appendage; ASD, atrial septal defect; LA, left atrium; RA, right atrium.

Previous studies have indicated that procedural success proportion in LAA closure clinical trials is 88–98.5% (24–27) and PFO/ASD clinical trials is 97.9–100% (28, 29). The surgical complexity of combining PFO/ASD occlusion and LAA closure whatever simultaneous or staged is more difficult than single closure. Our study indicated that combination had a procedural success proportion similar to the single closure, which may suggest the feasibility of combining PFO/ASD occlusion and LAA closure. One important determinant for closure success is operator experience. LAA closure has to be considered a complex interventional procedure with a relatively flat learning curve (30, 31). There is a significant improvement in procedural success with increased operator experience. Another important determinant is from puncturing the atrial septum or through a PFO/ASD during access to LAA. Transseptal puncture is almost always successful in experienced hands. However, when facing difficult anatomies, it carries the clinical risks in less experienced hands (32). Accessing the left atrium through a PFO has historically been discouraged mainly because of the concern that the PFO is located too high for delivery sheath, but Koermendy et al. using a PFO for left atrial access (17), while Kuwata et al., Kleinecke et al. and Wang et al. used PFO/ASD for access (33). These studies approved that the PFO/ASD facilitates left atrial access, and hence can be used as default path. Furthermore, this avoids the potential complications of transseptal puncture (34, 35). Based on our experiences of LAA and ASD closure, using transesophageal echocardiography or transthoracic echocardiography to define the individual ASD/PFO anatomy would be smooth to perform left atrial access (Figures 3A–D). However, cardiac computed tomography angiography also offers unique imaging planes, which allows for preplanning anatomic assessment, and post-procedure follow-up.

Both procedures are known to have side-effects (AF and device-related thrombus for PFO closure, device-related thrombus and iatrogenic atrial septal defect for LAA closure). The source of complications could develop from operating to device. Our study shows the similar complication rate in combining procedural closure when comparing with LAA closure. Death, cardiac tamponade, device embolization, major bleeding and stroke occurrence were not significantly different than the reference standard. Surprisingly, our study indicates that cardiac tamponade occurrence reached high among other complications in combination closure. Pericardial effusion was the most common complications in LAA closure (36). Rapid fluid accumulation in the pericardium can lead to cardiac tamponade, which is rare but life-threatening. The causes of cardiac tamponade are thought to be related to perforation of the atrial and aortic wall as well as oversizing of the device (37, 38), which are best avoided with meticulous technique and thorough use of imaging modalities. It should be noted that a limitation of this combining procedure is that the device embolization, dislocation during the peri-procedural period cannot be quickly treated. The protocol of Gafoor et al. is to wait 5–10 min after LAA occlude placement before closing the PFO/ASD to maintain access to the left atrium. A second limitation of this combining procedure is a social economic one. For example, according to the German medical policy, the LAA closure and PFO/ASD closure could not be done at the same time (21). It is thus more prudent to close the LAA first and then perform ASD closure at a subsequent session.

Thrombus formation is a recognizable and potentially harmful complication. Dukkipati et al. and Fauchier et al. showed that thrombus formation on devices was strongly associated with a higher risk of strokes during follow-up (39, 40). In contrast, Jacqueline Saw et al. showed that device-associated thrombus was not associated with increased risk for thromboembolism (41). Thus, the occurrence of device-related thrombus (DRT) may have device, implantation, or patient-specific risk factors, and repeat transesophageal echocardiography or computed tomographic angiographic imaging should be performed, as was recommended for management of DRT (42). The occurrence of iatrogenic atrial septal defects is common after LAA closure, particularly persistent iatrogenic atrial septal defects. Although the available data of iatrogenic atrial septal defects associated with adverse clinical events is scarce and inconclusive, it should be considered as a possible cause of stroke.

Our investigation demonstrated that combining PFO/ASD and LAA closure had low events incidence in follow-up, however, the finding could not simply determine the combining procedural efficacy due to the complexity of this topic. Firstly, there are many different scenarios-the preexist LAA closure alone adding PFO or ASD closure; the preexist PFO or ASD closure alone adding LAA closure; concurrent LAA closure and PFO or ASD closure-making the duration and postprocedural anticoagulation between the closures should be considered. Secondly, ASD and PFO are totally different in terms of pathophysiology and outcome (e.g., ASD is more for hemodynamic consequences while PFO is more related to paradoxical embolism), thus separate analysis of the setting of ASD and PFO would be preferable. Thirdly, there was no specific recommendation on LAAC given in the 2014 American guidelines, the 2016 European guidelines for the management of AF provided a class IIb recommendation for percutaneous LAAC in patients with AF and contraindications for long-term OAC, based on data from the PROTECT-AF and PREVAIL trials, the only LAAC randomized trials to date. Thus, the indications for the patients who performed closures before the newest recommendation were concerned. Meanwhile, the data extraction of the studies included in our systemic and meta-analysis was not enough to analyze different scenarios and different malformations of the septum. The investigation of combining procedural efficacy of our article should therefore be used as a call for further research to determine the best procedural strategy for patients indicated combining LAA closure and PFO or ASD closure.

There are several limitations of our study. The data offered in majority of the studies are retrospective with inherent disadvantages. Therefore, selection bias was evident. Secondly, the number of included patients is limited and the small studies are liable to introduce unstable results. Thirdly, the closure devices, follow-up duration, and post procedure antithrombotic regimen are different among these studies, which might affect the accuracy of the pooled estimates.

## 5. Conclusion

Although this systematic review and meta-analysis demonstrate the technical feasibility and safety of combining closure of PFO/ASD and LAA, further studies of sufficient sample size, long-term follow-up, and rigor endpoint criteria are yet needed to fully evaluate this combination procedure for its role in clinical outcomes.

## Data availability statement

The original contributions presented in this study are included in the article/Supplementary material, further inquiries can be directed to the corresponding author.

## Author contributions

YS contributed to the conception, design of the study, and wrote the first draft of the manuscript. YS, HX, and PK organized the database and performed the statistical analysis. All authors contributed to manuscript revision, read, and approved the submitted version.
