# Supplementary material for: The feasibility and safety of combining atrial septal defect/patent foramen ovale and left atrial appendage closure: A systematic review and meta-analysis
Source: Front Cardiovasc Med. 2023 Jan 6;9:1080257. doi: 10.3389/fcvm.2022.1080257 (PMC9854394; doi:10.3389/fcvm.2022.1080257)
Supplement: Supplementary file 1 [file Table_1.docx]

Supplementary Table S1 Clinical Characteristics

| Study/Year | Procedural workups | | | | LAA device | ASD device | PFO device | Post procedure antithrombotic regimen | Follow-up duration (m) |
| --- | --- | --- | --- | --- | --- | --- | --- | --- | --- |
|  | Before | Intra | Post | Follow-up |  |  |  |  |  |
| Dezsoe Koermendy 2014 | TEE | Fluo­roscopy  Angiography | TTE | TEE | ACP | ASO, ACSO | APO, ACSO | Acetylsalicylic acid 100 mg and clopidogrel 75 mg for 1–6 months | 14.5±9.7 |
| Sameer Gafoor 2014 | - | Fluo­roscopy Angiography  TEE | - | - | PLAATO, Watchman | Amplatzer | | Aspirin indeﬁnitely and clopidogrel for 3– 6 months | - |
| Jianming Wang 2018 | - | Fluo­roscopy  Angiography  TEE | TEE | TEE, TTE | LACBES, AAO | SHSMA | SHSMA | Clopidogrel 75 mg and aspirin 100 mg for 6 months then aspirin 100 mg indeﬁnitely | 11.8±3.0 |
| Shingo Kuwata 2018 | TEE | Fluo­roscopy  Angiography | TTE | TEE | ACP, AAO, ASO, APO | | | Clopidogrel 75 mg for 1–3 months and acetylsalicylic acid 100 mg for 5–6 months | 24.0±9.6 |
| Jiangtao Yu 2019 | TEE | TEE  Angiography | TTE | TEE | IrisFIT  Figulla FlexII | Amplatzer | Amplatzer | Warfarin, or combined enoxaparin with aspirin to Warfarin till 45 days | 6 |
| Caroline Kleinecke 2020 | - | - | - | TEE | ACP | - | - | Immediate OAC cessation if previously administered, acetylsalicylic acid for at least five, and clopidogrel for 1–6 months | 4–6 |
| Ming Chern Leong 2020 | - | Fluo­roscopy  TEE | TEE | TEE | Watchman | - | - | Aspirin indefinitely | 32.6±2.8 |
| Xiaofei Jiang 2020 | Angiography  TEE | Fluo­roscopy  Angiography  TEE | - | TEE TTE | Watchman | - | - | Rivaroxaban 15 mg for 45 days after which clopidogrel 75 mg and aspirin 100 mg for 6 months, then evaluate the patient's basic cardiovascular disease and give single antiplatelet or not | 3 |
| Zhi-hui Zhang 2020 | TTE  TEE | TEE Angiography | - | TEE TTE | - | - | - | Low-molecular-weight Heparin, Oral warfarin or Novel Oral Anticoagulants(NOAC) was administered at 45–60 days | 12 |
| Xiaofei Jiang 2021 | TEE | TEE | TEE | TEE | Watchman | - | - | Rivaroxaban 15 mg for 45 days after which clopidogrel 75 mg and aspirin 100 mg for 6 months | 12 |

TEE, transesophageal echocardiography; TTE, transthoracic echocardiography; ACP, Amplazer cardiac plug; AAO, Amplatzer amulet occluder; ASO, Amplatzer septal occluder; ACSO, Amplatzer cribriform septal occlude; APO, Amplatzer PFO occluder; N/A, Not applicable.
