# Supplementary material for: The feasibility and safety of combining atrial septal defect/patent foramen ovale and left atrial appendage closure: A systematic review and meta-analysis
Source: Front Cardiovasc Med. 2023 Jan 6;9:1080257. doi: 10.3389/fcvm.2022.1080257 (PMC9854394; doi:10.3389/fcvm.2022.1080257)
Supplement: Supplementary file 2 [file Table_2.docx]

| **Study/Year** | **Patient selection criteria** |
| --- | --- |
| **Dezsoe Koermendy 2014** | Indications for LAAC comprised of a history of previous bleeding, patients at high-risk of bleeding, unstable INR, high risk or history of falls, or patient preference, closure of the PFO or ASD was generally performed for primary or secondary prevention. |
| **Sameer Gafoor 2014** | Indications for LAAO based on a history of AF and inability to take anticoagulation or patient desire to be off of anticoagulation. ASD was closed according to guidelines for congenital heart disease. PFO was closed for cryptogenic stroke. |
| **Jianming Wang 2018** | Inclusion criteria: (1), Patients with non-valvular AF, age ≥ 18 years, CHA2DS2VASc ≥ 1. (2), Patients having a contraindication to anticoagulant therapy or unwilling to receive long-term warfarin therapy, HASBLED ≥ 3. (3), PFO/ASD closure based on current recommendations. (4), Patients can tolerate aspirin and clopidogrel.  Exclusion criteria: (1), Must long-term use of warfarin for anticoagulation. (2), Patients with valvular heart disease, initial AF, paroxysmal AF with a clear cause. (3), Suspected thrombus or confirmed thrombus in heart. (4), New York Heart Association class IV, left ventricular ejection fraction < 30%. (5), Patients with new-onset infections, tumor, haemorrhage, or ischemic stroke/TIA. (6), Patients with contraindictions of transesophageal echocardiography. |
| **Shingo Kuwata 2018** | The patients (≥ 40 years) undergoing percutaneous closure of a large ASD (≥ 20 mm) or a PFO with large atria (left atrium [LA] ≥ 40 mm, parasternal long axis) and at the same time of the LAA. Patients neither had clinical or electrocardiographic signs of prior AF nor an AF associated embolic ischemic or bleeding event. In the patients with prior ischemic events, AF had been excluded by long-term electrocardiogram (ECG). The LAA closure was performed for primary prevention. |
| **Jiangtao Yu 2019** | The inclusion and exclusion criteria for LAAC was based on European guidelines. The PFO/ASD closure was because of a large right-to-left shunt (>30 MBs). At the meantime, the brain MRI was performed in a patient with PFO. The ROPE scores were calculated in these patients. |
| **Caroline Kleinecke 2020** | Indications for LAAC was in line with current recommendations, a wide range of AF patients with estimated high thromboembolic risk who had contraindications for OAC were included. Indications for PFO/ASD closure based on current recommendations. |
| **Ming Chern Leong 2020** | The patients with ASD and chronic AF who were suitable for transcatheter occlusion were included. Suitable subjects were defined as patients who had no significant coronary artery disease or cardiac pathology, which would otherwise obviate them from having a transcatheter procedure. Patients who had a significant concomitant cardiac pathology that may have benefited from a cardiac surgery where all the pathologies can be treated in the same setting were excluded. All subjects underwent CHADS2, CHA2DS2-VASc, and HAS-BLED score assessment to evaluate the risk of thrombotic strokes and bleeding on Warfarin prior to the procedure. |
| **Xiaofei Jiang 2020** | Inclusion criteria: (1), Secundum ASD with diameter of 5-36 mm, the distance from the edge of the defect to the coronary sinus, the superior and inferior vena cava and the pulmonary vein ≥ 5 mm, and the atrioventricular valve ≥ 7 mm. (2), Patients with non-valvular AF persistent > 1 year. (3), Age > 18 years. (4), CHA2DS2-VASc ≥ 2. (5), PFO/ASD closure based on current recommendations.  Exclusion criteria: (1), Suspected thrombus or confirmed thrombus in heart. (2), Maximal LAA ostial width > 31 mm or < 17 mm. (3), Left ventricular function damaged. (4), Must long-term use of warfarin for anticoagulation. |
| **Zhi-hui Zhang 2020** | Inclusion criteria: (1), For patients with PFO, they have ischemic stroke, or transient ischemic attack (TIA), or a peripheral thromboembolic event or a large right-to-left shunt. For patients with ASD, TTE show clear indications for ASD occlusion. (2), TEE show clear indications for LAAC. (3), Non-valvular AF. (4), CHA2DS2VASc score ≥ 2, and HAS-BLED score ≥ 3 or having a contraindication to anticoagulant therapy or unwilling to receive long-term warfarin therapy. (5), Patients were willing to accept the one-stop occlusion.  Exclusion criteria: (1), Valvular heart disease. (2), Left atrial or LAA thrombosis. (3), ASD requiring surgical repair, congenital heart diseases requiring other thoracotomy treatment. (4), Severe heart failure (New York Heart Association class IV). (5), Severe renal or hepatic insufficiency. (6), Acute stroke occured within1 month. (7), Patients were unwilling to accept the one-stop occlusion. |
| **Xiaofei Jiang 2021** | Inclusion criteria: (1), PFO with right to left shunt of Ⅲ-Ⅳ (contrast transcranial Doppler). (2), Secundum ASD with diameter of 5-36 mm, the distance from the edge of the defect to the coronary sinus, the superior and inferior vena cava and the pulmonary vein ≥ 5 mm, and the atrioventricular valve ≥ 7 mm. (3), Patients with non-valvular AF persistent. (4), Age > 45 years. (5), CHA2DS2-VASc ≥2. (6), PFO/ASD closure based on current recommendations.  Exclusion criteria: (1), Implantation of mechanical valves or moderate to severe mitral stenosis. (2), Suspected thrombus or confirmed thrombus in heart. (3), Maximal LAA ostial width > 31 mm or < 17 mm. (4), Left ventricular ejection fraction < 35%. (5), Must long-term use of warfarin for anticoagulation. |

Supplementary Table S2 Patient selection criteria

LAAC, left atrial appendage closure; INR, international normalized ratio; PFO, Patent foramen ovale; ASD, Atrial septal defect; AF, atrial fibrillation; TTE, transthoracic echocardiography.
